# Small but Heavy Role: MicroRNAs in Hepatocellular Carcinoma Progression

**DOI:** 10.1155/2018/6784607

**Published:** 2018-05-22

**Authors:** Erbao Chen, Xiaojing Xu, Ruiqi Liu, Tianshu Liu

**Affiliations:** Department of Medical Oncology, Zhongshan Hospital, Fudan University, Shanghai, China

## Abstract

Hepatocellular carcinoma (HCC), which accounts for 85–90% of primary liver cancer, is the fifth most common malignant tumor and the third leading cause of cancer-related deaths worldwide, but the pathological mechanism of HCC is still not fully elucidated. miRNAs are evolutionarily endogenous small noncoding RNAs that negatively regulate gene expression via posttranscriptional inhibition or target mRNA degradation in several diseases, especially human cancer. Therefore, discovering the roles of miRNAs is appealing to scientific researchers. Emerging evidence has shown that the aberrant expressions of numerous miRNAs are involved in many HCC biological processes. In hepatocarcinogenesis, miRNAs with dysregulated expression can exert their function as oncogenes or tumor suppressors depending on their cellular target during the cell cycle, and in tumor development, differentiation, apoptosis, angiogenesis, metastasis, and progression of the tumor microenvironment. In this review, we summarize current findings on miRNAs and assess their functions to explore the molecular mechanisms of tumor progression in HCC.

## 1. Introduction

As a major type of primary liver cancer (85–90%), hepatocellular carcinoma (HCC) is one of the most notoriously invasive and aggressive human malignancies around the world and causes 500,000–600,000 deaths every year. Based on the high fatality of liver cancer (overall mortality-to-incidence ratio, 0.95), this type of epithelial cancer is still a major global health burden [1]. Despite the advancements in the diagnosis and therapy of HCC, the prognosis remains unsatisfactory due to high rates of relapse and metastasis. Etiologically, the development of HCC is prominently associated with fibrosis and cirrhosis, which is mainly caused by chronic hepatitis B virus (HBV) and/or hepatitis C virus (HCV) infection [2]. Other pathogenic reasons are diabetes [3, 4], obesity [5], intake of aflatoxin B1 [6], alcohol abuse [7], nonalcoholic fatty liver disease [8], and metabolic disease [9]. With exposure to these high risks, the liver suffers a series of hyperplastic and dysplastic disease stages and ultimately acquires a malignant phenotype with intrahepatic metastasis and extrinsic hepatic foci. Cancer progression is thought to involve the disordered regulation of oncogenes and tumor suppressor genes that have greatly influenced the advancement of knowledge on the molecular mechanisms of HCC development in certain processes such as the cell cycle, angiogenesis, apoptosis, and cell migration and metastasis. Therefore, the identification of different cellular factors and a clear understanding of the underlying molecular mechanisms that are involved in the development of tumor metastasis are critical.

As previously described, miRNAs can significantly contribute to the criteria. Substantial functional studies reveal molecular cellular phenomena linked to miRNAs as well as apoptosis, inflammation, cell cycle, and the tumor microenvironment in concert with various oncocytic molecular events facilitate tumor development, progression, and metastasis [10]. MiRNAs, a new class of short small (average 23 nucleotides in length) highly conserved noncoding RNAs, negatively control the expression of many target genes in HCC [11]. In addition, computational analyses show that miRNAs can regulate more than one-third of protein-coding gene expression transcriptionally and posttranscriptionally [12]. Thousands of miRNAs that bind to target mRNA through 3′-untranslated region (UTR) interactions have been identified. On the basis of data from a vast majority of miRNAs, aberrant oncogene activation and/or tumor suppressor inactivation commonly lead to HCC. Clarifying the complex characteristic of miRNAs could provide a clearer perception of HCC. In recent years, there has been a considerable advancement in the understanding of the role of miRNAs in cancer.

In this paper, we will summarize the role of miRNAs in hepatocarcinoma and in the progression of HCC, focusing on their regulatory roles as tumor suppressors and oncogenes in networks involving metastatic hepatocellular cancer.

## 2. Brief Introduction of miRNA Biogenesis

MiRNA genes are usually located in endonuclear noncoding regions, such as introns of coding genes. These genes are transcribed to shape hundreds of nucleotide long primary transcripts (pri-miRNAs) with RNA polymerase II, capped with 7-methylguanosine and polyadenylated. Subsequently, these pri-miRNAs are processed in the nucleus by the RNase III enzyme Drosha in assistance with its cofactor, the DiGeorge syndrome critical region gene 8 (DGCR8 also known as Pasha), into dozens of base-pair precursor miRNA (pre-miRNAs), which constitute a partly corresponding short hairpin structure [13]; then they are transported into the cytoplasm with assistance from the RAS-related nuclear protein through the RNA GTP-dependent transporter Exportin 5 (XPO-5). RNase III endoribonuclease Dicer and its partner HIV-1 transactivate response RNA binding protein (TPBP) then guide pre-miRNAs into mature miRNAs composed of 20–24 nucleotides in the cytoplasm, which subsequently constitute the RNA-induced silencing complex (RISC) [14]. The miRNA/RISC complex then binds to the 3′-UTR of target mRNAs and regulates their expression depending on the level of base-pair matching [12]. RISC degrades target mRNA by perfect pairing with mRNA 3′-UTR. Otherwise, RISC inhibits target mRNA translation by imperfect pairing with mRNA 3′-UTR.

## 3. Dysregulation of miRNAs in HCC

MiRNAs are affirmed by pivotal regulatory functions and regulate many cellular biological processes; alterations in their expression contribute to neoplasm [84]. Many studies have revealed numerous miRNAs changes in HCC by multifarious microarray analysis, which can compare differential miRNA expression profiles between tumor and tumor-adjacent tissues, normal and cancer tissues, primary tissues and metastatic/recurrent foci, or miRNA expression differences induced by anticancer treatment. In addition, quantitative RT-PCR is often used in validating miRNA expression in clinical specimens. Many studies have reported several miRNAs related to hepatitis virus, differentiation, cell cycle, apoptosis, angiogenesis, invasion, and metastasis. These studies are summarized in Table 1.

## 4. Aberrant miRNAs in the Cell Cycle

Many studies show that dysregulation in the cell cycle is an important step in the development of hepatocarcinogenesis and miRNAs participate in this process. MiR-199a/b-3p can target tumor-promoting PAK4 to suppress HCC growth through inhibiting PAK4/Raf/MEK/ERK pathway [15]. MiR-302b suppresses HCC hyperplasia due to associating with proliferation-related proteins, such as AKT2, CCND1, and CDK2, targeting the EGFR/AKT2/CCND1 pathway [16]. In addition, miR-302b suppresses HCC cell proliferation by targeting EGFR [17]. Ectopic expression of miR-184 restrains SOX7 protein, resulting in upregulation of c-Myc and Cyclin D1 expression and the phosphorylation of Rb to promote cell proliferation, tumorigenicity, and cell cycle progression [18]. MiR-200a suppresses the proliferation of HCC cells by induction of G1 phase arrest by CDK6 [19]. MiR-19a inhibits cell growth by targeting Cyclin D1 [20]. Notably, miR125a-5p and miR-125b are upstream regulators of oncogenic sirtuin 7 (SIRT7), which is always overexpressed in HCC, and loss of SIRT7 induced p21WAF1/Cip suppresses cycle D1 expression, thus increasing the G1/S stage as well as suppressing proliferation [21]. The suppressor of variegation 3-9 homolog 1 (SUV39H1), the prototype of histone methyltransferase, is a major enzyme element for histone H3 lysine 9 trimethylation. The expression of miR-125b inhibits HCC development and metastasis by downregulating endogenous SUV39H1 expression at both messenger RNA and protein levels [22]. Interestingly, miR-140 inhibits hepatocellular genesis and arrests the cell G2/M phase by repressing TGFB receptor 1 (TGFBR1) and fibroblast growth factor 9 (FGF9) signaling through mitogen-activated protein kinase/extracellular signal-regulated kinase (MAPK/ERK) signaling [23]. Cancer cells can escape from the immune system to evade apoptosis and retain survival in the cancer microenvironment through many ways. CCNE1, CDC25A, CCND3, CDK4, and BTRC have been identified as direct targets of miR-497 and miR-195, which leads to aberrant cell proliferation in hepatocarcinogenesis [24]. The expression of miR-145 could induce G(2)–M cell cycle arrest and apoptosis through targeting multiple components of oncogenic insulin-like growth factor (IGF) signaling, including insulin receptor substrate- (IRS1-) 1, IRS2, and insulin-like growth factor 1 receptor signaling pathway [85]. MiR-451 inhibits the tumorigenicity of cells associated with the downregulation of cyclin D1 and c-Myc, through targeting direct suppression of IKK-beta [25]. MiR-494 is overexpressed in human HCC tissues and joins in transformation by regulating the G1/S cell cycle transition through targeting mutation suppressors in colorectal cancer tumors [86]. The deregulation of miR-517a and miR-517c promotes HCC cell proliferation via targeting Pyk2 [26]. MiR-371-5p facilitates the G1/S transition cell cycle by downregulating pre-mRNA processing factor 4 homolog B (PRPF4B) in human HCC cells [27]. MiR-221 overexpression is related to the status of tumor capsular infiltration in HCC clinical samples, and miR-221 inhibits cell growth and apoptosis-mediating G1/S-phase arrest [28]. MiR-221 also suppresses the p53 protein combining with MDM2 [29].

Overexpression of miR-1269 accelerates cell proliferation in HCC aiding in directly suppressing FOXO1 (MAPK/ERK) signaling [30]. MiR-148a expedites cell proliferation, cell cycle progression, cell migration, and anchorage independent growth by downregulating the PTEN protein [31]. MiR-1228 exerts pleiotropic function as an oncogene by promoting the cell cycle and cell mobility and negatively regulating the expression of p53. Downregulation of p53 expression conversely increases expression of miR-1228, thereby acting as a positive feedback loop that contributes to hepatogenesis in HCC [32].

MiR-138 can regulate cyclin D3 and function as an oncogene in HCC [33]. Repression of hepatocyte nuclear factor-3beta (HNF-3beta) by miR-141 suppresses hepatocellular cell proliferation and invasion and promotes apoptosis [34]. By degradation of the sex-determining region Y- (SRY-) box 7 (SOX7), overexpression of miR-24 promotes the proliferation and invasion of neoplasm cells [35]. Overexpression of miR-133a attributes to suppression of HCC cell growth by inhibition of Akt activation and the ERK signal pathway [36].

## 5. Aberrant miRNAs in Apoptosis

MiR-129-5p could inhibit the degradation of IkappaB-alpha, increase apoptosis, and reduce the migration of HCC cells by suppressing the valosin-containing protein (VCP) [37]. Overexpression of miR-122 diminishes the capacity of Galpha12 to increase the c-Met pathway to activate ERK, STAT3, and Akt/mTOR, suppressing cell proliferation by augmenting apoptosis [38, 39]. Similarly, through the c-Met/PI3K/Akt pathway, miR-93 promotes cell proliferation, migration, and invasion and also allows liver cancer cell to avoid apoptosis by directly inhibiting PTEN and CDKN1A expression [40]. In the same way, miR-105 suppresses cell proliferation by downregulating insulin receptor substrate-1, 3-phosphoinositide-dependent protein kinase-1, and AKT1 directly [41]. Estrogen activates miR-23a and p53 expression via estrogen receptor-alpha transcriptionally to control apoptosis in liver neoplasms [87]. Both let-7g and let-7i, mediated by the Bcl-xL protein, deliver a concurrent effect to curb hepatoma cell proliferation and apoptosis [88]. MiR-125b promotes apoptosis by suppressing the antiapoptotic molecules Mcl-1, Bcl-w, and IL-6R [42]. MiR-34a decreases mRNA protein stability to increase the expression of beta-catenin through degradation of long intergenic noncoding RNA UFC1 [43]. Forced overexpression of miR-144 remarkably reduces cell proliferation, increases apoptosis, and suppresses migration and invasion of HCC cells [89]. A proliferation-inducing ligand (APRIL), a member of the tumor necrosis factor (TNF) superfamily, is a newly discovered target of miR-383, contributing to tumor apoptosis [44]. MiR-34a suppresses cell growth, migration, and invasion; meanwhile, it increases cellular apoptosis and caspase activity in HCC cells [50]. Cells with high expression of miR-106a have stronger invasive ability, quicker cell cycle progression, and less apoptosis compared with the low expression cell line [90]. MiR-221 exerts inhibitory effect on apoptosis by targeting Bmf. Patients who have miR-221 overexpression are associated with a more lethal pathological pattern and less time to recurrence after surgery [45]. The data described above are summarized in Table 2.

## 6. Aberrant miRNAs during Invasion and Metastasis

Invasion and metastasis, two important features of tumors, are the most lethal reasons for neoplasm relapse and poor prognosis [10]. Some patients stem from a background of chronic hepatic diseases like fibrosis. One study found that miR-125b functions with an inhibitory effect on epithelial-mesenchymal transition (EMT) and EMT-associated signaling pathway by targeting small mothers against decapentaplegic (SMAD) 2 and 4 [91]. MiR-181a has a critical role in inducing hepatocyte EMT, which is a good substitute for the TGF-*β*-induced effect. VASH2 expression mediated by miR-200 in HCC cells expedites the malignant transformation of liver tumors by inducing EMT [46]. MiR-200a functions as an important regulatory inhibition factor in EMT by targeting the beta-catenin pathway [47]. CDC42, CDH1, PAK2, and BCL-2 are validated as targets of miR-224, which exhibits increased expression of both mRNA and protein levels in HCC cells as well as in HCC tissues [48]. MiR-122, which comprises around 70% of the liver's total miRNAs, promotes hepatic fibrogenesis by targeting Kruppel like factor (KLF6); moreover, restoration of miR122a reduces the cancer incidence in mice through inhibiting EMT [49]. MiR-451 inhibited cell growth, induced G0/G1 arrest, and promoted apoptosis. Importantly, miR-451 can inhibit EMT [92].

MiR-329 significantly regulates cell invasion by targeting bromodomain containing 4 (BRD4) but has no effect on cell proliferation and apoptosis [51]. MiR-362-5p works through CYLD to activate the NF-kappaB signaling pathway to increase cell proliferation, clonogenicity, migration, invasion, and metastasis, which are associated with HCC progression [52].

Vascular invasion provides a direct route for tumor metastasis. MiR-494 can trigger gene silencing of multiple invasion-suppressor miRNAs by inhibiting genomic DNA demethylation by direct targeting of TET1, thereby leading to tumor vascular invasion. Metastasis is a complex process that involves multiple steps. By downregulating the antitumor genes tensin homology protein (PTEN) and cyclin-dependent kinase inhibitor 1 (CDKN1A), miR-93 can promote cell proliferation, migration, and invasion by activating the c-Met/PI3K/Akt signaling pathway; additionally, anti-miR-93 transfection makes HCC cells more sensitive to sorafenib and tivantinib treatment [40]. Phosphatase and tensin homolog (PTEM) also is validated as a functional target of miR-216a/217. In combination with PTEM and decapentaplegic homolog 7 (SMAP7), miR-216a/217 raises the stem-like cell population and promotes the metastatic ability of HCC cells through activating the PI3K/Akt and TGF-*β* pathways [53, 54]. MiR-221 modulates the tumor suppressor HDAC6 through coordinated JNK/c-Jun and nuclear factor kappa B (NF-kappaB) signaling during HCC development and progression [55]. MiR-657 directly binds to the transducin-like enhancer protein 1 (TLE1) 3′-UTR and contributes to hepatic carcinogenesis by activating NF-kappaB pathways [56]. NF-kappaB is a critical factor linking inflammation and metastasis. MiR-195 exerts its suppressive function by decreasing the expression of multiple NF-kappaB downstream effectors by directly targeting IKK alpha and TAB3 [57]. MiR-331-3p promotes cancer cell proliferation and EMT-mediated metastasis through suppression of the PH domain and leucine-rich repeat protein phosphatase-mediated dephosphorylation of Akt [58]. Some tumor suppressor miRNAs exert their functions in multiple ways. Restoration of miR-7 inhibits HCC cell growth, migration, and metastasis through regulating the phosphoinositide 3-kinase (PI3K)/Akt pathway targeting phosphoinositide 3-kinase catalytic subunit delta (PIK3CD), rapamycin (mTOR), and p70S6K [59, 60]. The data described above are summarized in Table 3.

## 7. Aberrant miRNAs in Angiogenesis

MiR-214 contributes to cancer cell apoptosis, cell cycle, and angiogenesis by mediating the hepatoma-derived growth factor (HDGF) [61]. Overexpression of miR-125b in HCC cells decreases placenta growth factor expression and regulates the angiogenesis index [62]. MiR-29b exerts its antiangiogenesis function by suppressing matrix metalloproteinase-2 (MMP-2) expression [63]. Expression of human sulfatase-1 (hSulf-1) and PTEN mediated by miR-21 lead to activation of Akt/ERK pathways and EMT in HCC cells and finally enhance the activity of HCC cell proliferation and movement and promote HCC xenograft tumor growth [64]. Through activating the PIK3C2alpha/Akt/HIF-1alpha/VEGFA pathway, miR-26a acts in the degradation of vascular endothelial growth factor A (VEGFA) to modulate angiogenesis [65]. MiR-195 also prevents tumor angiogenesis and metastasis by interacting with vascular endothelial growth factor (VEGF) and the prometastatic factors VAV2 and CDC42 [66]. FGF2 and VEGFA are the two most potent angiogenic factors. MiR-503 deters neoplasm angiogenesis via targeting FGF2 and VAGFA [67]. Upregulation of miR-146a strengthens the angiogenic activity of endothelial cells by stimulating platelet-derived growth factor receptor-alpha (PDGFRA) [68]. MiR-126-3p contributes to metastasis and angiogenesis in HCC due to degradation of LRP6 and PIK3R2 [69]. MiR-26a reduces tumor angiogenesis of HCC through hepatocyte growth factor-cMet (HGF-cMet) signaling [70]. MiR-302c suppresses tumor growth in HCC through inhibition of EMT [71]. By inhibiting vascular endothelial growth factor A (VEGFA) and astrocyte elevated gene-1 (AEG-1), miR-497 suppresses microvessel densities in xenograft cancers [72]. Interestingly, there is a negative feedback loop between polycomb repressive complex (PRC2) and miR-101 that affects the progression and metastasis of HCC. MiR101 is repressed by PRC2 through c-Myc signaling, although in contrast, the expressions of EZH2 and EED, the two subunits of PRC2, are inhibited by miR-101 [73]. In essence, these results suggest a balancing act between tumor suppressors and oncogenes. Another study shows miR-148a-5p functions by directly targeting and inhibiting Myc expression, whereas miR-363-3p destabilizes Myc protein by directly targeting and inhibiting USP28. However, inhibition of miR-148a-5p or miR-363-3p induces hepatocarcinoma by promoting G1–S-phase cell cycle transition, whereas their activation has the opposite effect [74]. MiR-139 interacts with Rho-kinase 2 (ROCK2) and reduces its expression in HCC cells line. Downregulation of miR-139 improves the invasive capacity of HCC cells in vitro and HCC metastasis in vivo [75]. MiRNA-188-5p suppresses cancer cell proliferation and metastasis in vitro and in vivo by interacting with fibroblast growth factor 5 (FGF5) [76].

## 8. Role of miRNAs in the Microenvironment 

Exosome-mediated miRNA transfer is an important mechanism of intercellular communication in HCC cells [93]. MiR-375 suppresses hepatoma cell growth targeting astrocyte elevated gene-1 (AEG-1) [77]. MiR-122 affects the metabolism of carcinoma cells through pyruvate kinase M2 [78]. It demonstrates that STAT3 signaling is a core pathway mediating human immune suppression in the tumor microenvironment. Micro-146a, an important downregulator of immune activity, exerts a negative effect on tumor development in HCC cells by promoting the expression of STAT3 activation-associated cytokines, like TGF-*β*, IL-17, VEGF, and I IFN, leading to HCC-induced NK cell dysfunction [79]. Abnormal autophagy activity decreases miRNA-224 targeting of Smad4 to accumulate cell migration and tumor formation [80, 81]. Enhanced TGF-*β* activity suppresses the expression of miR-34a, leading to a high production of chemokine CCL22, which recruits regulatory T (Treg) cells to facilitate cancer cell immune escape [82]. Li et al. demonstrated that miR-148a expression was significantly lower in a cancer stem cell-like subtype, which is clinically aggressive and associated with poor survival, than in other subtypes. Moreover, the miR-148a–ACVR1-BMP-Wnt circuit could profile/determine an advanced clinical stem cell-like subtype of HCC [83]. The data described above are summarized in Table 4.

## 9. Conclusions

Sustained dysregulation of miRNAs, some of which act as oncogenes or tumor suppressors, plays an unequivocal role in the development of HCC. Its role in HCC development and metastasis has been clearly reported and supported by evidence from various lines of studies [33]. MiRNAs function as a new tiny class of gene regulators at the translation and/or posttranscriptional level. They exhibit different expression patterns in various types of cancers [34]. The dysregulation of miRNAs has been demonstrated to play a significant effect on cell cycle, apoptosis, invasion, migration, and metastasis in HCC through the interaction of cellular signaling networks. In the past three years, lots of progress has been made in understanding the accurate mechanisms for aberrant miRNA profiles; meanwhile, more targets and upstream regulatory molecules of these dysregulated miRNAs have been reported. In this review, we summarize the role of miRNAs in carcinogenesis and progression of HCC and supply a more distinct comprehensive picture of their status in gene interaction signaling pathways. Collectively, the investigative studies have resulted in a better understanding of cancer-related miRNA functions and their roles as tumor suppressors and oncogenes. Given the implication of a large number of miRNAs in the control of key tumor suppressors and oncogenes, the deregulation of specific miRNAs has been shown to greatly influence HCC development, invasiveness, prognosis, and treatment response. From a diagnostic point of view, high stability of miRNAs in circulation makes them perfect biomarkers.

However, many studies observe abnormally expressed miRNAs in HCC, associated with the identification of miRNA clinical signatures, which are correlated with HCC progression or metastasis; this makes them potential diagnostic and/or prognostic markers. One of the biggest challenges is that miRNA clinical signatures are always validated in small samples. Therefore, larger specimens should be used to test precisely the hundreds of miRNAs in future studies. Nevertheless, using these miRNAs to achieve clinical diagnosis and treatment remains a continuous challenge to science researchers.

## Figures and Tables

**Table 1:** Processes involved in hepatocarcinogenesis and associated microRNAs.

Processes	target	microRNAs
Cell cycle and apoptosis	Cyclin D	miR-199a/b-3p, miR-19a, miR-451, miR-138, miR-302b
	p53	miR-1228, miR-221, miR-1228
	PTEN	miR-148a, miR-93, miR-216a/217, miR-21
	SOX7	miR-184, miR-24
	c-myc	miR-451, miR-148a-5p, miR-363-3p
	AKT	miR-133a, miR-105, miR-331-3p
	Bcl-2	miR-125b, miR-224, miR-200
Invasion and metastasis	SMAD	miR-125b, miR-224
	Beta-catenin	miR-200a
	MMP-2	miR-29b
	PIK3	miR-126-3p, miR-7
angiogenesis	VEGF	miR-195, miR-146a
	VEGFA	miR-302c, miR-26a
	AEG-1	miR-375, miR-302c

PTEN, phosphatase and tensin homolog; SOX7, SRY-related HMG-box; MMP-2, matrix metalloproteases 2; VEGF, vascular endothelial growth factor; and AEG-1, astrocyte elevated gene-1.

**Table 2:** Aberrant miRNAs associated with the cell cycle and apoptosis.

MicroRNAs	Type of deregulation	Target	Reference
miR-199a/b-3p	Downregulated	Cyclin D1	[15]
miR-302b	Downregulated	EGFR, Cyclin A, Cyclin D1, CDK2	[16, 17]
miR-184	Upregulated	SOX7	[18]
miR-200a	Downregulated	CDK6	[19]
miR-19a	Downregulated	Cyclin D1	[20]
miR125a-5p, miR-125b	Downregulated	sirtuin7, SUV39H1	[21, 22]
miR-140	Downregulated	TGFBR1, FGF9	[23]
miR-497, miR-195	Upregulated	CCNE1, CDC25A, CCND3, CDK4,	[24]
miR-451	Downregulated	cyclin D1, c-Myc	[25]
miR-517a, miR-17c	Upregulated	Pyk2	[26]
miR-371-5p	Downregulated	PRPF4B	[27]
miR-221, miR-1228	Upregulated	p53	[28, 29]
miR-1269	Upregulated	FOXO1	[30]
miR-148a	Upregulated	PTEN	[31]
miR-1228	Upregulated	p53	[32]
miR-138	Upregulated	Cyclin D3	[33]
miR-141	Downregulated	HNF-3beta	[34]
miR-24	Upregulated	SOX7	[35]
miR-133a	Upregulated	AKT, ERK	[36]
miR-129-5p	Downregulated	VCP	[37]
miR-122	Upregulated	Galpha12	[38, 39]
miR-93	Upregulated	PTEN, CDKN1A	[40]
miR-105	Upregulated	AKT1	[41]
miR-125b	Upregulated	Bcl-w, IL6R	[42]
miR-34a	Upregulated	UFC1	[43]
miR-383	Downregulated	APRIL	[44]
miR-221	Downregulated	Bmf	[45]

**Table 3:** Aberrant miRNAs associated with invasion and metastasis.

MicroRNAs	Type of deregulation	Target	Reference
miR-200	Upregulated	VASH2 CDC42, CDH1, PAK2, BCL-2	[46, 47]
miR-224	Downregulated	CDC42, CDH1, PAK2, BCL-2	[48]
miR-122	Downregulated	KLF6	[49]
miR-200a	Upregulated	Beta-catenin	[50]
miR-329	Upregulated	BRD4	[51]
miR-362-5p	Upregulated	CYLD	[52]
miR-93	Upregulated	PTEN, CDKN1A	[40]
miR-216a/217	Upregulated	PTEM, SMAP7	[53, 54]
miR-221	Upregulated	HDAC6	[55]
miR-657	Upregulated	TLE1	[56]
miR-195	Downregulated	IKK alpha, TAB3	[57]
miR-331-3p	Upregulated	AKT	[58]
miR-7	Downregulated	PIK3CD	[59, 60]

**Table 4:** Role of miRNAs in the tumor microenvironment.

MicroRNAs	Type of deregulation	Target	Reference
miR-214	Upregulated	HDGF	[61]
miR-125b	Upregulated	placenta growth factor	[62]
miR-29b	Downregulated	MMP-2	[63]
miR-21	Downregulated	hSulf-1, PTEN	[64]
miR-26a	Downregulated	VEGFA	[65]
miR-195	Downregulated	VEGF, VAV2, CDC42	[66]
miR-503	Downregulated	FGF2, VAGFA	[67]
miR-146a	Upregulated	PDGFRA	[68]
miR-126-3p	Upregulated	LRP6, PIK3R2	[69]
miR-26a	Downregulated	c-MET	[70]
miR-302c	Downregulated	VEGFA, AEG-1	[71]
miR-497	Downregulated	PRC2	[72]
miR101	Downregulated	EZH2, EED	[73]
miR-148a-5p/miR-363-3p	Downregulated	MYC	[74]
miR-139	Downregulated	ROCK2	[75]
miR-188-5p	Downregulated	FGF5	[76]
miR-375	Downregulated	AEG-1	[77]
miR-122	Upregulated	pyruvate kinase M2	[78]
miR-146a	Upregulated	TGF-*β*, IL-17, VEGF, I IFN	[79]
miR-224	Upregulated	Smad4	[80, 81]
miR-34a	Downregulated	CCL22	[82]
miR-148a	Downregulated	ACVR1	[83]
